# Effects of Endotoxemia and Blood Pressure on Microcirculation and Noradrenaline Needs With or Without Dexmedetomidine in Beagle Dogs—A Blinded Cross-Over Study

**DOI:** 10.3390/ani15121779

**Published:** 2025-06-17

**Authors:** Barbara Steblaj, Fabiola Binia Joerger, Sonja Hartnack, Angela Briganti, Annette P. N. Kutter

**Affiliations:** 1 Section of Anaesthesiology, Department of Clinical Diagnostics and Services, Vetsuisse Faculty, University of Zurich, 8057 Zurich, Switzerland; 2 Section of Epidemiology, Vetsuisse Faculty, University of Zurich, 8057 Zurich, Switzerland; 3 Department of Veterinary Sciences, University of Pisa, 56124 Pisa, Italy

**Keywords:** alpha 2-adrenergic agonist, canine, norepinephrine, proportion of perfused vessels, sepsis, vasopressors

## Abstract

Endotoxemia often leads to microcirculatory derangement. In six sevoflurane anaesthetized Beagle dogs, we investigated the effects of 1 mg/kg of *Escherichia coli* lipopolysaccharide endotoxin intravenous and blood pressure (mean arterial pressure of 65 mmHg versus 85 mmHg) on microcirculation assessed on buccal mucosa using side stream dark field microscopy. Dogs were afterwards resuscitated with fluids and noradrenaline. We investigated dose requirements of noradrenaline with or without dexmedetomidine. Microcirculatory parameters, and markers of sepsis (cardiac output, mixed venous oxygen saturation, carbon dioxide gap, and lactate) were analysed before endotoxemia, after endotoxemia, after a 30 mL/kg of Ringer’s acetate fluid bolus, and during noradrenaline +/− dexmedetomidine infusion, after a second fluid bolus, and a second time after vasopressor treatment in a cross-over fashion. Endotoxemia and mean arterial pressure had no statistically significant effect on microcirculation; however, endotoxemia resulted in a decrease in cardiac output. Dexmedetomidine neither improved microcirculation nor reduced noradrenaline requirements.

## Simple Summary

Endotoxemia often leads to a drop of systemic blood pressure and subsequently deranged microcirculation. This needs to be aggressively addressed with the use of fluids and, if needed, supported by cardiovascular supportive drugs with vasoconstrictive properties, e.g., noradrenaline. As dexmedetomidine also induces vasoconstriction, its use could potentially reduce the dose of noradrenaline. Additionally, some animal studies suggest that dexmedetomidine may improve microcirculatory derangements induced by endotoxemia. However, little data is available on microcirculation in endotoxemic dogs. In this study we investigated the effects of endotoxemia and blood pressure on microcirculation and noradrenaline requirements with or without dexmedetomidine in six sevoflurane anaesthetised Beagle dogs. Using side stream dark field microscopy, we assessed microcirculatory parameters on buccal mucosa and markers of sepsis. After intravenous injection of endotoxin and consequent cardiovascular collapse, dogs were hemodynamically stabilised with fluids and noradrenaline with or without dexmedetomidine (cross-over). Endotoxemia resulted in no changes in microcirculation. Dexmedetomidine did not improve microcirculation or reduce noradrenaline requirements. Markers of sepsis showed alterations. Early and aggressive treatment of endotoxemia might prevent microcirculatory derangements.

## 1. Introduction

Endotoxemia and septicemia are conditions with consequences on macrocirculation, microcirculation, coagulation cascade, intestinal permeability, renal and other internal organ function. Consequently, organ failure can ultimately lead to death [1]. Early goal directed therapy has been proposed by Rivers et al. [2], as it supposedly led to better survival. However, PROMISE [3] and TRIAL [4] studies have questioned early goal-directed therapy, as the survival did not improve compared to standard protocols, despite improvements in macrocirculatory parameters such as cardiac output. The question remains whether early goal directed therapy also results in an optimised microcirculation.

The surviving sepsis campaign guidelines, 2016 [5] and 2021 [6], have suggested treatment algorithms for sepsis. Fluid resuscitation at 30 mL/kg should be administered in the first 3 h and mean arterial pressure (MAP) should be targeted to ≥65 mmHg. If the MAP goal cannot be achieved, the first-choice vasopressor is noradrenaline. In endotoxemic dogs, noradrenaline has been shown to result in better organ function, systemic perfusion, and survival compared to adrenaline [7], and in increased renal blood flow compared to non-endotoxemic dogs [8]. These studies suggest that noradrenaline might be the most suitable drug of choice also in canine endotoxemia.

As endotoxemia can result in a systemic inflammatory response, a large part of research in recent decades has been focused on how to decrease systemic inflammation and reduce detrimental organ damage. Dexmedetomidine, a highly specific alpha-2 adrenergic agonist, has been shown to reduce mortality [9] and inhibit the systemic inflammatory response during endotoxemia in rats [10] and to reduce inflammation in dogs with ventilator-induced lung injury [11]. Moreover, dexmedetomidine has been shown to downregulate inflammatory responses [12], to maintain or increase microcirculation [13], and to protect against intestinal epithelial barrier disruption [10] in rat endotoxemia. Dexmedetomidine also improved the microcirculation post-operatively in humans following cardiac surgery [14]. In septic hamsters, sepsis-evoked microcirculatory derangements were attenuated by dexmedetomidine [12]. Additionally, it has been demonstrated that alpha-2 agonists diminish noradrenaline requirements in septic humans [15], sheep [16], and rats [13]. In other words, less noradrenaline was needed to achieve the targeted blood pressure. On the other hand, dexmedetomidine and other alpha-2 agonists, can produce hemodynamic changes, which might result in negative alterations in microcirculation.

The aim of this study was to report if endotoxemia induces changes on microcirculatory parameters and whether a constant rate infusion (CRI) of dexmedetomidine exerts beneficial effects on microcirculation and leads to a reduction in noradrenaline requirements. As a secondary aim we wanted to assess if a higher target MAP of ≥85 mmHg would improve microcirculatory parameters. Therefore, noradrenaline was administered to initially achieve a MAP of 65 mmHg, followed by a subsequent target of 85 mmHg.

We hypothesised that endotoxemia will negatively affect microcirculatory parameters and that dexmedetomidine will improve or maintain microcirculatory parameters and result in a reduction of noradrenaline requirements. The second hypothesis was that a MAP ≥ 85 mmHg would further improve microcirculatory parameters.

## 2. Materials and Methods

Ethical committee approval was obtained by Swiss authorities, Canton Zurich (ZH244/17). Study was designed as a blinded, randomised, cross-over study. The CRIs were prepared by person, not involved in measurements. Randomisation was performed online (www.randomizer.org, last accessed 25 May 2018).

Six purpose-bred Beagle dogs (3 intact females and 3 intact males) aged 7.4 (5–9.8) years (median (range)) with a body weight of 13.7 (11.4–17.9) kg were included in this study. The dogs had been scheduled for euthanasia due to the primary need of autopsy for another terminal parasitology study, where the presence of *Dirofilaria repens* in the skin (between 176 and 221 days after the treatment) was investigated (non-published work for pharmaceutical company).

Based on a pre-anaesthetic clinical examination, complete blood cell count, urine, blood gas and serum biochemistry analysis, all dogs were found to be healthy and showing no systemic effects of parasitic infestation. Before undergoing this study, the dogs were included in a magnetic resonance imaging study [17]. Afterwards, the current study was performed. The fluid responsiveness [18], coagulation [19], and kidney parameters [20] of the current study have been published previously.

### 2.1. Anaesthesia, Instrumentation, and Measurements

All dogs had a full physical examination prior to anaesthesia. A venous catheter (Vasofix^®^ 20G; BBraun, Provet, Lyssach, Switzerland) was aseptically placed in a cephalic vein and 0.2 mg/kg methadone (Methadon Streuli^®^; Streuli Pharma AG, Uznach, Switzerland) was administered intravenously (IV). Anaesthesia was induced with 2 mg/kg propofol (Propofol 1% Fresenius Kabi AG, Kriens, Switzerland) IV bolus and additionally titrated to effect. Dogs were orotracheally intubated with a cuffed endotracheal tube (Rüschelit^®^ Super Safety Clear; Teleflex Medical, Belp, Switzerland) and sevoflurane (end-tidal concentration 1.5–1.8 vol%, Sevorane; AbbVie AG, Cham, Switzerland) in oxygen and air (inspiratory fraction of oxygen of 0.6) was administered. Dogs were placed in lateral recumbency and artificially ventilated (Avance S 5; Anandic Medical Systems AG, Feuerthalen, Switzerland) with pressure-controlled ventilation (PCV), where maximum pressure equalled 8–12 cm H_2_O and respiratory rate was adjusted to maintain end-tidal carbon dioxide (EtCO_2_) between 35 and 45 mmHg. Ringer’s acetate (Ringer Acetat; Fresenius Kabi AG, Kriens, Switzerland) was administered at 5 mL/kg/h. Oesophageal temperature (T°) was maintained between 37.5 °C and 39 °C with a forced air warming unit (3 M™ Bair Hugger™ 505; Photon Surgical Systems Ltd., Quedgeley, UK). Fentanyl was administered at 5 mcg/kg/h.

An 8.5-Fr introducer and an 8-Fr × 110 cm Swan-Ganz pulmonary artery catheter (746-F8, Swan Ganz Thermodilution Catheter, Edwards Lifesciences, Nyon, Switzerland) were placed in the right jugular vein, a central venous catheter (Blue Flex Tip Catheter, Arrow International, Teleflex Medical GmbH, Belp, Switzerland) in the left jugular vein, an arterial catheter (Vasofix^®^ 20G; BBraun, Provet, Lyssach, Switzerland) in metatarsal artery, and a urinary catheter (Portex, 2.6 mm OD, 50 cm; Smiths Medical, Adliswil, Switzerland) in the urethra. All catheters were placed after clipping and aseptical preparation of all catheter insertion sites as described elsewhere [18]. At all time points, before macro- and microcirculatory parameters were evaluated, mixed venous and arterial blood samples were withdrawn in pre-heparinized syringes and analysed immediately (Rapid Point^®^ 500, Siemens Healthineers, Zurich, Switzerland). From these results the carbon dioxide (CO_2_) gap was calculated as mixed venous partial pressure of CO_2_ (PmvCO_2_)—arterial partial pressure of CO_2_ (PaCO_2_). At each time point, the following parameters were measured: heart rate (HR) from electrocardiogram (ECG), pulse-oximetry, EtCO_2_, and blood T°. Arterial, pulmonary, right atrial, and pulmonary artery occlusion pressure and cardiac output (CO) by thermodilution were measured, as described previously [18]. Thereafter, the side stream dark field (SDF) camera (MicroScan, MicroVision Medical, Amsterdam, The Netherlands) was placed on the buccal mucosa (above the left upper canine tooth) that was gently cleaned of secretions with an isotonic-saline drenched gauze sponge. The probe was placed in just slight contact with mucosa and overpressure was avoided. Only after good quality images were obtained, at least 3 consecutive videos were recorded. Thereafter, the measurements were repeated at the sublingual mucosa. Automatically analysed parameters (using AVA 4.3 software, MicroVision Medical, The Netherlands) were Perfused de Backer density (pDBD) and Proportion of perfused vessels (PPV) in all vessels (<100 μm) and in vessels < 20 μm. Vessels < 20 μm included arterioles, capillaries and venules, which have a diameter below 20 μm. The microvascular flow index (MFI) and heterogeneity index (HI) were manually assessed as described previously [21]. In short, the MFI was quantified by segmenting the video images into four quadrants. The predominant flow type within each quadrant was evaluated using a standardised scoring system (0 = absent flow, 1 = intermittent flow, 2 = sluggish flow, 3 = normal flow) in accordance with the established consensus [22]. The overall MFI for the video was obtained by calculating the mean value across all four quadrants. Additionally, the HI, which serves as an indicator of blood flow heterogeneity and reflects distributive abnormalities [23], was computed using the formula: HI = (MFI_maximum_ − MFI_minimum_)/MFI_mean_.

**Figure 1:**
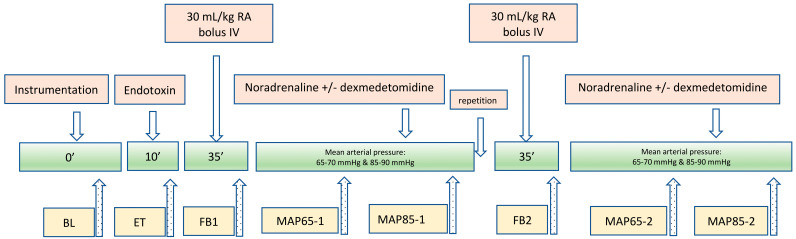
Flow chart of study design in 6 sevoflurane anaesthetised Beagle dogs.

A comprehensive summary of all interventions and measurements is presented in Figure 1.

After baseline measurements (BL), 1 mg/kg of *Escherichia coli* lipopolysaccharide (LPS) (O111:B4; Sigma-Aldrich, Taufkirchen, Germany) [8] diluted with 20 mL 0.9% saline (NaCl 0.9%, Dr. G. Bichsel, Interlaken, Switzerland) was infused IV over 10 min. Thereafter macro- and microcirculation measurements were repeated (ET). Afterwards, the dogs were resuscitated with 30 mL/kg of Ringer’s acetate bolus administered IV over 30 min. Measurements of macro- and microcirculatory parameters were repeated thereafter (FB1). Dexmedetomidine (Dexdomitor, Orion Pharma, Zug, Switzerland) constant rate infusion (CRI) at 0.5 mcg/kg/h or an equal volume of NaCl 0.9% was started in a cross-over design. Administration of noradrenaline (Noradrenaline Sintetica; Sintetica, Mendrisio, Switzerland) was started 10 min later at the rate of 0.1 mcg/kg/min. Noradrenaline was titrated in increments of 0.1 mcg/kg/min every 5 min to achieve the targeted MAPs. Upon reaching an infusion rate of 0.6 mcg/kg/min, the rate was increased by 0.2 mcg/kg/min. After reaching a dose of 2.2 mcg/kg/min, subsequent increments of 0.4 mcg/kg/min were administered every 5 min. Once the MAP of 65 mmHg and 85 mmHg were achieved, the measurements were repeated (MAP65-1 and MAP85-1). Thereafter, both infusions were stopped and after a 10 min washout period the study protocol was repeated, i.e., a bolus of 30 mL/kg of Ringer’s acetate (FB2) and afterwards titration of noradrenaline with dexmedetomidine or NaCl 0.9% to achieve the target MAPs of 65 mmHg and 85 mmHg, respectively (MAP65-2 and MAP85-2).

After achieving MAP 85 mmHg, and measurements of macro- and microcirculatory parameters were recorded, the dogs were euthanized while still in anaesthesia with a 0.5 mL/kg bolus of a product containing embutramide (T61, MSD, Lucerne, Switzerland) IV.

### 2.2. Statistics

Descriptive statistics and graphs were performed using Prism 9 (GraphPad Software Inc., San Diego, CA, USA). Data were analysed using a linear mixed-effects model fit by restricted maximum likelihood performed by the statistical software R version 3.6.2 (R Core Team 2020) and the package nlme [24]. Missing data was imputed with the package Miss forest. Period 1 was defined as the time between FB1 and MAP85-1 and Period 2 as the time between FB2 and MAP85-2. Dog was used as a random effect and microvascular parameters, period, treatment (dexmedetomidine or NaCl), and time point as fixed effect. The goodness-of-fit metric was based on Akaike information criterion (AIC) with a lower AIC indicating a better model fit (a difference of at least 2). Microvascular parameters were analysed at time ET, MAP 65 and MAP 85, and ET compared to baseline. A *p*-value of < 0.05 was considered as statistically significant.

## 3. Results

Due to too many missing values for sublingual, only the buccal mucosal microvascular data is presented and was analysed statistically. In a Supplementary Table (Supplementary Table S1), data on microcirculatory parameters measured at the sublingual site are available.

**Figure 2:**
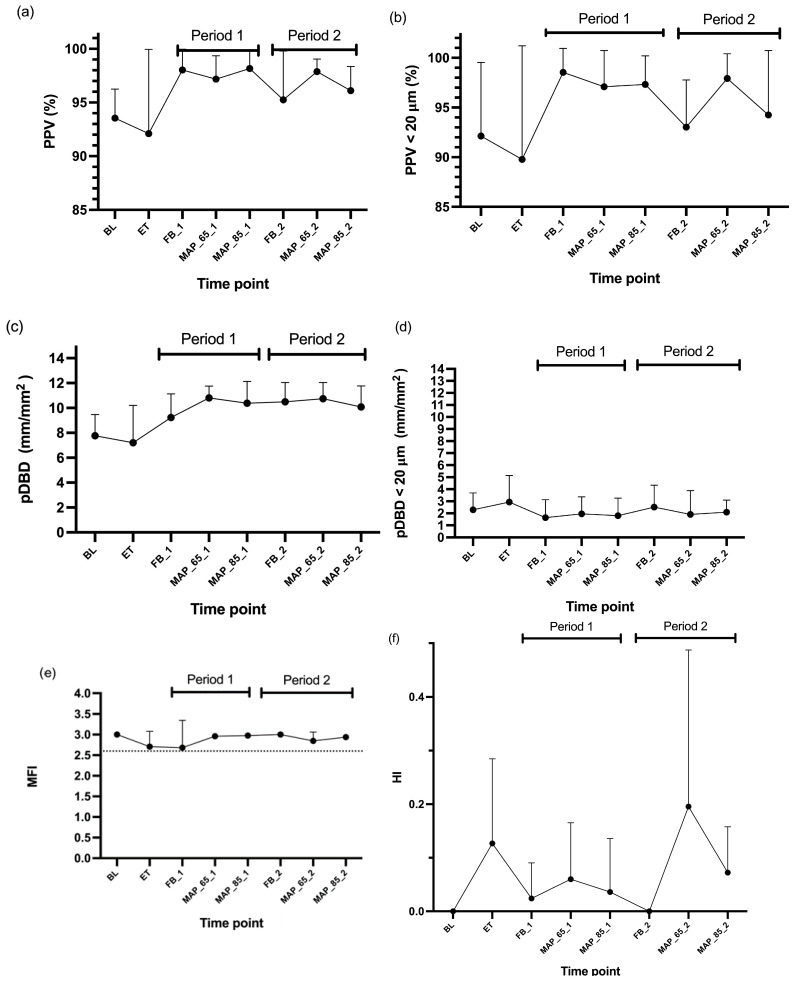
Mean and standard deviations of (**a**) Proportion of perfused vessels, (**b**) Proportion of perfused vessels < 20 µm, (**c**) Perfused de Backer Density, (**d**) Perfused de Backer Density < 20 µm, (**e**) Microvascular flow index, (**f**) Heterogeneity index in six Beagles at eight time points measured at buccal mucosa. Legend: BL, baseline; ET, endotoxin; FB_1, fluid bolus (Period 1); MAP_65_1, mean arterial pressure of 65–70 mmHg (Period 1); MAP_85_1, mean arterial pressure of 85–90 mmHg (Period 1); FB_2, fluid bolus (Period 2); MAP_65_2, mean arterial pressure of 65–70 mmHg (Period 2); MAP_85_2, mean arterial pressure of 85–90 mmHg (Period 2); PPV, Proportion of perfused vessels; pDBD, perfused de Backer Density; MFI, Microvascular flow index; HI, Heterogeneity index. Dotted line represents the value of 2.6 for MFI.

**Figure 3:**
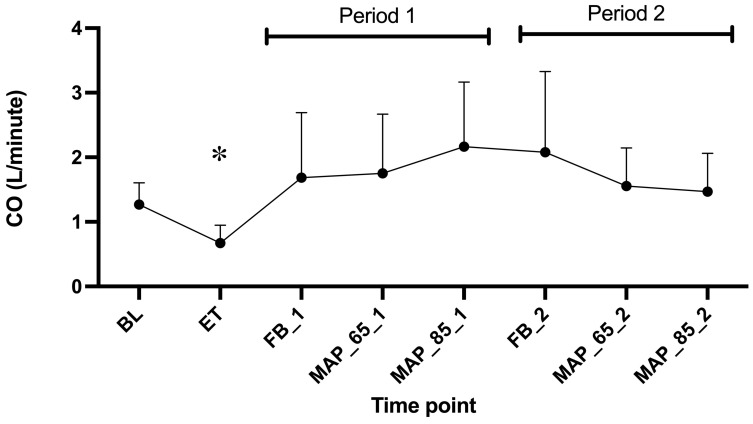
Mean and standard deviations of cardiac output in six Beagles at eight time points. Legend: BL, baseline; ET, endotoxin; FB_1, fluid bolus (Period 1); MAP_65_1, mean arterial pressure of 65–70 mmHg (Period 1); MAP_85_1, mean arterial pressure of 85–90 mmHg (Period 1); FB_2, fluid bolus (Period 2); MAP_65_2, mean arterial pressure of 65–70 mmHg (Period 2); MAP_85_2, mean arterial pressure of 85–90 mmHg (Period 2); CO, cardiac output. Asterisk (*) presents a time point significantly different to the baseline.

The values for PPV, PPV < 20 μm, and pDBD appeared to return to levels above baseline following the first fluid bolus, based on observed data trends (Figure 2a–c). Descriptive statistics indicated that the same occurred in CO (Figure 3). The values for pDBD < 20 μm (Figure 2d) seemed to behave differently and remained low. Descriptive analysis indicated that the values for MFI and HI appeared not to be restored following the administration of the initial fluid bolus (Figure 2e,f).

Period and treatment with dexmedetomidine had no statistically significant effect on any of the microcirculatory variables. There was also no significant effect of endotoxemia on baseline values of any microvascular parameter (Table 1 and Figure 2), but there was a significant effect on CO (Table 1). There was no effect of MAP 85 mmHg versus 65 mmHg on any microcirculatory parameter or cardiac output (Table 2 and Figure 2 and Figure 3).

Only CO was significantly lower after induction of endotoxemia (Table 1, Figure 3).

**Figure 4:**
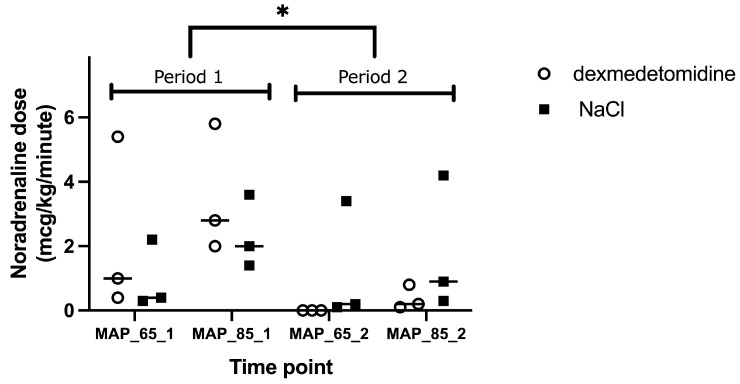
Individual maximal dose of noradrenaline dose with or without dexmedetomidine constant rate infusion in six Beagles at four time points. Legend: MAP_65_1, mean arterial pressure of 65–70 mmHg (Period 1); MAP_85_1, mean arterial pressure of 85–90 mmHg (Period 1); MAP_65_2, mean arterial pressure of 65–70 mmHg (Period 2); MAP_85_2, mean arterial pressure of 85–90 mmHg (Period 2). Dots represent dexmedetomidine 0.5 mcg/kg/hour constant rate infusion and full squares infusion of NaCl. Asterisk (*) presents a significant difference in noradrenaline dose between Periods 1 and 2.

There was no difference in noradrenaline dose with or without dexmedetomidine CRI (*p* = 0.97). However, period influenced noradrenaline dose, with less noradrenaline being needed in Period 2 (*p* < 0.001, Figure 4).

**Figure 5:**
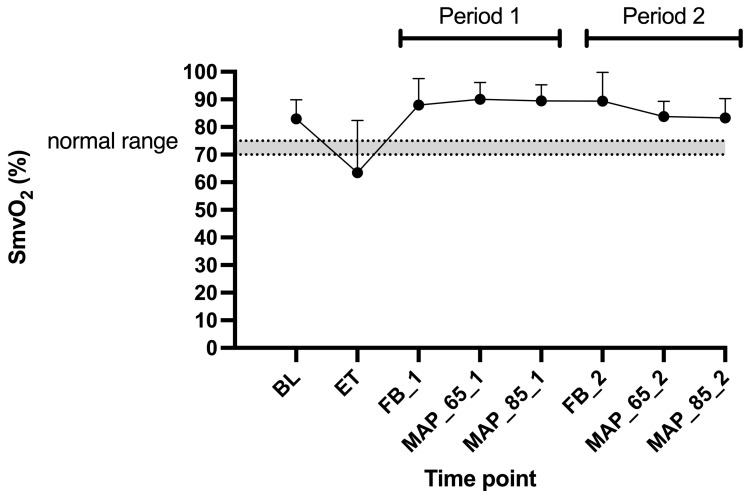
Mean and standard deviations of mixed-venous oxygen saturation in six Beagles at eight time points. Legend: BL, baseline; ET, endotoxin; FB_1, fluid bolus (Period 1); MAP_65_1, mean arterial pressure of 65–70 mmHg (Period 1); MAP_85_1, mean arterial pressure of 85–90 mmHg (Period 1); FB_2, fluid bolus (Period 2); MAP_65_2, mean arterial pressure of 65–70 mmHg (Period 2); MAP_85_2, mean arterial pressure of 85–90 mmHg (Period 2); SmvO_2_, mixed-venous oxygen saturation. Normal reference range for SmvO_2_: 70–75%, which is marked as grey area within dotted lines.

**Figure 6:**
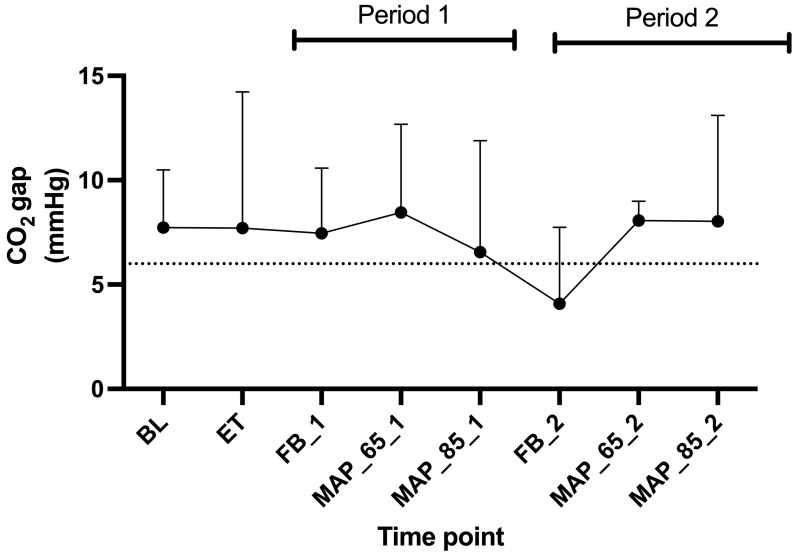
Mean and standard deviations of the CO_2_ gap (mixed-venous - arterial partial pressure of CO_2_) in six Beagles at eight time points. Legend: BL, baseline; ET, endotoxin; FB_1, fluid bolus (Period 1); MAP_65_1, mean arterial pressure of 65–70 mmHg (Period 1); MAP_85_1, mean arterial pressure of 85–90 mmHg (Period 1); FB_2, fluid bolus (Period 2); MAP_65_2, mean arterial pressure of 65–70 mmHg (Period 2); MAP_85_2, mean arterial pressure of 85–90 mmHg (Period 2); PmvCO_2_, mixed-venous CO_2_ pressure; PaCO_2_, arterial CO_2_ pressure. Dotted line presents a PmvCO_2_-PaCO_2_ difference of 6 mmHg.

**Figure 7:**
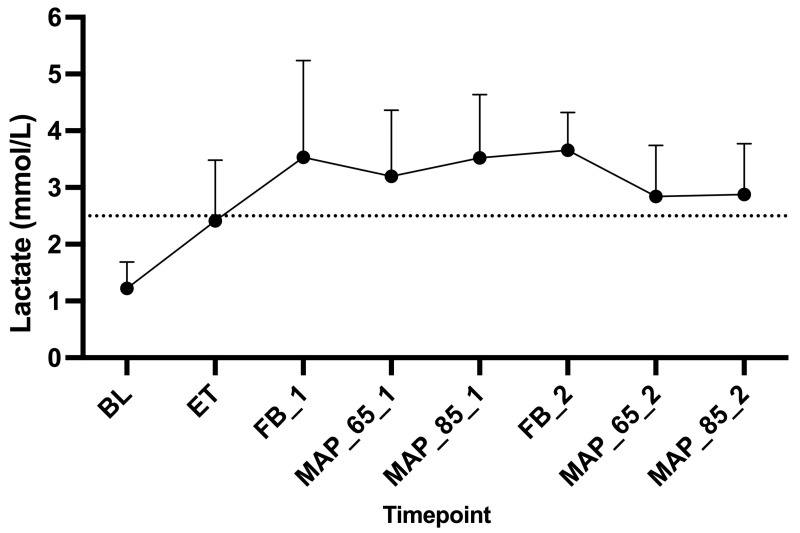
Mean and standard deviations of lactate measured in arterial blood sample in six Beagles at eight time points. Legend: BL, baseline; ET, endotoxin; FB_1, fluid bolus (Period 1); MAP_65_1, mean arterial pressure of 65–70 mmHg (Period 1); MAP_85_1, mean arterial pressure of 85–90 mmHg (Period 1); FB_2, fluid bolus (Period 2); MAP_65_2, mean arterial pressure of 65–70 mmHg (Period 2); MAP_85_2, mean arterial pressure of 85–90 mmHg (Period 2); dotted line represents upper reference value of 2.5 mmol/L.

Descriptive data of blood gas results are presented in Figure 5, Figure 6 and Figure 7: mixed-venous oxygen saturation (SmvO_2_) decreased below 70% during endotoxemia and was restored after the FB1 to high normal values (Figure 5).

The CO_2_ gap was very variable (Figure 6).

Lactate began to increase at ET and remained elevated during all remaining time points (Figure 7).

The statistical analysis revealed that neither SmvO_2_, CO_2_ gap, nor lactate were significantly affected by endotoxemia or dexmedetomidine.

## 4. Discussion

In this cross-over study in six Beagle dogs, endotoxemia did not significantly alter the assessed microcirculatory variables. However, endotoxemia resulted in a well described decrease in CO. Additionally, dexmedetomidine neither had a significant effect on microcirculatory variables, nor reduced the noradrenaline requirements significantly. A MAP of 85 mmHg also had no significant effect on microvascular variables.

### 4.1. Microcirculatory Parameters

Although we could not show in the current study in six dogs that endotoxemia significantly affected microvascular variables, several human studies have demonstrated that sepsis negatively alters microcirculation [25,26,27,28]. Direct comparison between our study and clinical human studies is difficult, due to the species, study settings, and site of measurement.

#### 4.1.1. PPV

Peruski et al. [29] performed a study in anaesthetized dogs using SDF imaging and reported lower baseline buccal PPV (82.7 ± 8.3% (mean ± SD) than we measured in our study (95 ± 7.4%). They used oxymorphone, diazepam, and ketamine, while we used methadone and propofol for induction of anaesthesia. In another study in our institution with a very similar anaesthesia protocol to the current study baseline, PPV was 93 ± 5.6% [30]. Maintenance was in both studies with sevoflurane and fentanyl CRI. In the study of Peruski et al. where celiotomy was performed, it is likely that end-tidal sevoflurane values were higher. It is known that sevoflurane negatively affects PPV in people [31]. Additional differences between the above studies were also the fluid choice. We administered Ringer’s acetate and Peruski infused Ringer’s lactate. A recent study (Gruell et al. in preparation), demonstrated that administration of Ringer’s lactate resulted in lower PPV values than the administration of acetate-containing Plasma Lyte A. In our study, the first measurement was performed after some maintenance dose of Ringer’s acetate had already been administered during instrumentation.

#### 4.1.2. PPV < 20 μm

In studies in septic humans, sublingual PPV < 20 μm was much lower, with 48 (33–61)% [25] and 72 (43–94)% [32], compared to our study, where buccal PPV < 20 μm was 96 (85–100)%. However, other studies in septic humans [28,33] demonstrated similar results to ours. These differences are likely to arise from the individuals’ status and site of measurements. We chose to measure at the buccal and sublingual site. The sublingual site proved to be difficult, which led to missing data. Therefore, only the buccal measurements were analysed. Values of buccal PPV < 20 μm in our study were high (above 85%), which likely reflects that endotoxemia produced little effect on microcirculation at this site. This outcome may be attributed to our study design, which involved the administration of endotoxin rather than assessing naturally occurring sepsis, where bacterial endotoxin release persists, until it is mitigated by antibiotic treatment. Additionally, we corrected the volemic status of dogs quickly with fluid bolus. This likely led to a better PPV.

#### 4.1.3. MFI and HI

The interpretation of microvascular flow relies significantly on experience and expert knowledge. However, it is well-established and intuitively recognised that reduced flow, along with increased flow variability, are both indicative of microcirculatory dysfunction. An MFI ≤ 2.6 indicates a state of microcirculatory alteration [23]. Our results for MFI (2.7 (2.0–3.0)) during ET were similar to results in former human studies during naturally occurring sepsis with the values: 2.92 (2.59–3.0) [28], 2.7 (1.5–3.0) [32], and 2.8 (2.6–2.97) [33], respectively.

The HI was 0.1 (0.0–0.36) during ET in our study. Similarly, in the human studies, HI in septic patients were reported to be 0.08 (0–0.43) [28], 0.07 (0–0.41) [32], and 0.18 (0.03–0.3) [33]. Pottecher et al. [34] reported baseline values in septic patients to be around 0.7 (0–3), which were much higher compared to other studies. However, when these patients underwent passive leg raising, values decreased significantly to 0.3 (0.1–1). This likely emphasises the dependency of HI values on the patient’s fluid status. Unfortunately, the values for elevated HI are not yet established in the human or veterinary literature.

We performed resuscitation after induced endotoxemia according to the surviving sepsis campaign [6] with fluid and noradrenaline administration. We tested the addition of dexmedetomidine CRI to noradrenaline CRI, as it has been shown that dexmedetomidine 100 μg/kg IV reduced needs for noradrenaline in rats and improved the microcirculation in septic conditions in hamsters [12]. Indeed, dexmedetomidine infusion resulted in beneficial microcirculation in the study of Miranda [12] in hamsters receiving 1 mg/kg *E. Coli* LPS and 5 mcg/kg/h dexmedetomidine. We administered the same dose of endotoxin, however with 10 times less dexmedetomidine. As rodents have higher drug metabolism and beneficial results were seen with 5 μg/kg/h, we assumed that a dose of 0.5 μg/kg/h in dogs would be sufficient, resulting in better microcirculation with minimal macrovascular effects. Whether increasing the dexmedetomidine dose would be beneficial remains to be investigated. Interestingly, in a recent study in dogs undergoing pyometra surgery [35], dexmedetomidine 3 μg/kg/h did not result in hemodynamic or microcirculatory compromise compared to fentanyl CRI. Additionally, Miranda et al. [12] states that for dexmedetomidine to decrease noradrenaline needs, a sufficient volume loading is needed. We assumed that 30 mL/kg fluid bolus would be sufficient for volume restoration after the used dose of LPS. Despite this, dexmedetomidine did not significantly reduce noradrenaline needs in our study. However, we found a significant difference between Period 1 and 2. We cannot exclude that the dogs were not sufficiently volume restored during Period 1 and this effect together with time from ET administration was more important than the effect of dexmedetomidine. Probably the non-significant changes in microcirculation were also too small for dexmedetomidine to show a significant effect, as also shown by the missing effect of endotoxin administration on microcirculatory parameters (Figure 2).

### 4.2. Marker of Sepsis

As microcirculation measurements have not yet been implicated as daily routine monitoring, we chose to additionally describe other markers of sepsis, which found their place in human monitoring in septic patients, i.e., CO, SmvO_2_, CO_2_ gap, and lactate.

#### 4.2.1. CO

We have observed a decrease in CO after endotoxin administration. After the administration of fluid boluses, the CO increased even above baseline values. This finding suggests that vasodilation and/or extravascular fluid loss was responsible for the drop in CO and that fluids successfully treated relative hypovolemia. The reason why CO increased above the baseline can likely be explained by the fact that the dogs were anaesthetized with inhalant anaesthetic, which are known to produce vasodilation. As we gave a bolus of 30 mL/kg, this probably refilled the vessels that were sub-optimally filled before the administration of endotoxin. The CO remained above baseline values until the end of the experiment. It has been shown in human sepsis studies that microcirculatory improvements and global circulatory effects following fluid administration can be achieved in early sepsis, but not late sepsis [36]. The current study would mimic early sepsis, and therefore fluid administration was probably effective.

#### 4.2.2. SmvO_2_ and CO_2_ Gap

We observed a drop of SmvO_2_ below 70% after the administration of endotoxin. This was expected as the body enters a disbalance between oxygen demand and oxygen delivery. This could partially be explained by a reduction in CO. The fluid boluses successfully restored the SmvO_2_ above 75% (Figure 5). We also calculated the CO_2_ gap between PmvCO_2_ and PaCO_2_, which provides real-time feedback. For most of the time, the CO_2_ gap remained above 6 mmHg. Opposed to SmvO_2_, the CO_2_ gap did not show any consistent changes.

#### 4.2.3. Lactate

Lactate levels increased above the upper reference value of 2.5 mmol/L after the administration of endotoxin and stayed at increased values throughout the study (Figure 7). Lactate is a marker often used in sepsis. The observed increase might have been as well affected by adrenergic stimulation [37]. Despite the cause, hyperlactatemia is consistently associated with severity of illness and prognosis [38]. As the lactate kinetics lag behind e.g., SmvO_2_ and CO_2_ gap, lactate should be combined with other markers of sepsis [39]. A flow chart was proposed by De Backer [40], which considers lactate, venous oxygen saturation (as a substitute for SmvO_2_), and CO_2_ gap. At ET, a lactate > 2 mmol/L, a CO_2_ gap > 6 mmHg and a venous saturation < 70% were measured in our study. According to DeBacker this would have led to a conclusion of a stage of low CO with dysoxia. In the current study, we indeed measured low CO values, which supports the use of the mentioned flow chart. After the endotoxin time point, the flow chart suggested a hemodynamic profile of microcirculatory alterations with dysoxia. Unfortunately, we could not statistically confirm microcirculatory alterations in our study, as we only assessed the impact of individual variables on the data set.

#### 4.2.4. MAP 65 mmHg Versus 85 mmHg

We chose to resuscitate blood pressure to two ranges of MAP (65 and 85 mmHg) in order to investigate potential differences in microcirculation between the two target blood pressures. We found no differences when comparing microvascular parameters at MAP of 65 mmHg and MAP of 85 mmHg. The SEPSIS SPAM study in people [41] comparing MAP of 65 mmHg and MAP of 85 mmHg also found no difference in 28-day mortality between the two groups; however, the incidence of newly diagnosed atrial fibrillation was higher in the MAP of 85 mmHg group. Therefore, surviving sepsis campaign guidelines [6] strongly recommend with moderate quality evidence a target MAP of ≥65 mmHg. The current study supports that a higher MAP target does not seem to improve the assessed parameters.

### 4.3. Limitations

The reason why we could not demonstrate significant changes in microvascular variables after the endotoxin administration might be the dose of endotoxin administered, the time frame, the sample size and the fact that this was not a clinical septic condition. The dose of endotoxin that we administered immediately produced a significant reduction in CO. However, we performed measurements directly after ET and thereafter quickly corrected CO with the administration of fluids and noradrenaline. Therefore, it might have been that more time without treatment would have been needed to observe changes in microcirculation after the administration of ET. Another possible explanation is that we firstly measured CO by repeated injection of 5% dextrose boluses, which may have influenced some of the microcirculatory parameters measured thereafter. Additionally, we only administered one bolus of endotoxin, which is eliminated with time. In clinical septic conditions, bacteria continuously release endotoxin, altering microcirculation until antibiotic therapy exerts its full effect. The two main objectives of this study were to answer whether dexmedetomidine exerts beneficial effects on microcirculation and if targeting MAP to 85 mmHg would have beneficial effects on microcirculatory parameters. We chose to use a cross-over design, which left us with only three dogs per group during one period receiving one of the treatments. However, to use a cross-over design was a good choice, as we found a significant period effect. If all dogs would have received dexmedetomidine or a sham treatment in the same order, we would have interpreted the data wrongly for dexmedetomidine based on the period effect. The main limitation is that we only had a small sample size. Due to a complex study design, and to answer the two main objectives of the study, we opted not to analyse the parameters with traditional statistics over the whole-time frame. We suggest that readers look at the data to make their own interpretation of overall values for each parameter. Another reason why we could not observe significant microvascular changes might lie in the initial inflammatory phase due to the injected endotoxin. This phase typically leads to vasodilation and inflammation, and it could have been that the vessels increased above 100 μm and were therefore no longer considered as microcirculatory network [27]. Additionally, our dogs were under general anaesthesia, which is known to affect the cardiovascular and respiratory system significantly. Anaesthesia could have affected our baseline values and also the following timepoints. However, we found it unethical to administer endotoxin in awake patients and the anaesthesia was kept as stable as possible during the study.

Another limitation of the study is that we did not give bolus of dexmedetomidine but started with CRI 10 min before treatment was initiated with noradrenaline. Afterwards, the time to achieve the targeted MAP ranged from 40 to 60 min. Due to the development of significant hemodynamic instability following endotoxin administration, we elected not to administer a loading bolus of dexmedetomidine. At this critical stage, further reductions in myocardial contractility and heart rate could have posed a severe risk to the animals’ survival. Current clinical recommendations for the use of dexmedetomidine in critically ill human patients also advise against bolus administration to mitigate the risk of profound bradycardia [42]. Accordingly, we selected the lowest CRI dose previously used in dogs [11], deliberately omitting the loading dose to avoid these adverse cardiovascular effects. It is acknowledged that steady-state plasma concentrations may not have been achieved within the timeframe of the present study. However, the reported elimination half-life of dexmedetomidine in healthy dogs appears to be relatively prolonged (28.28 ± 6.14 min) [43] compared to that observed in humans (~6 min) [44]. The extended half-life reported in canines may be partly attributed to methodological differences, particularly in sampling strategies, and may not be directly applicable to our study due to substantial differences in dosage regimens, which could influence both the pharmacokinetics and pharmacodynamics of the drug. Moreover, it is recognised that critical illness is often associated with reduced hepatic perfusion and diminished cardiac output, both of which can alter the pharmacokinetic profile of dexmedetomidine. Specifically, these factors may facilitate a more rapid attainment of stable plasma concentrations during CRI in critically ill patients. In the equine study, administering CRI of dexmedetomidine without a loading bolus, plasma concentrations increased rapidly and reached relatively stable levels within the first 30 min, coinciding with a pronounced increase in sedation. The half-life in this study was 20 min. This pharmacokinetic and pharmacodynamic profile, despite similar reported half-lives to those in dogs, closely parallels our clinical observations in critically ill canines receiving low-dose dexmedetomidine via CRI without an initial bolus. To date, however, there is a lack of published data on the pharmacokinetics of dexmedetomidine specifically in critically ill canine populations, which limits direct extrapolation.

Lastly acetated fluids that were administered during the current study were reported to induce vasodilation and decreased myocardial contractility in dogs [45,46]. As in the current study all dogs received the same fluids, the results are probably not influenced by the fluid choice.

## 5. Conclusions

Endotoxemia resulted in a significant decrease in CO. However, we could not show any significant effects of endotoxemia on microcirculatory parameters and therefore the effect of dexmedetomidine and different MAP targets could not be assessed with the current study. Dexmedetomidine at 0.5 mcg/kg/h did not reduce the noradrenaline dose significantly.

## Figures and Tables

**Table 1:** Effect of treatment dexmedetomidine versus NaCl, endotoxin versus baseline and Period 2 versus Period 1 on the microcirculatory parameters in six sevoflurane anaesthetised Beagle dogs.

Microcirculatory Variables (Buccal) and Cardiac Output	Effect of Treatment Dexmedetomidine Versus NaCl		Effect of Endotoxin Versus Baseline		Effect of Period 2 Versus Period 1	
	Estimated Effect	*p*-Value	Estimated Effect	*p*-Value	Estimated Effect	*p*-Value
Proportion of perfused vessels (%)	0.00	0.99	−0.02	0.77	−1.38	0.13
Proportion of perfused vessels < 20 µm (%)	−1.49	0.38	0.00	0.98	−2.58	0.07
Perfused DeBacker density (mm/mm^2^)	−0.16	0.75	0.01	0.95	0.31	0.49
Perfused DeBacker density < 20 µm (mm/mm^2^)	0.44	0.47	0.09	0.58	0.37	0.45
Microvascular flow index (no unit)	0.01	0.92	−0.14	0.43	0.06	0.51
Heterogeneity index (no unit)	0.05	0.39	0.19	0.30	0.05	0.28
Cardiac output (L/minute)	−0.31	0.08	2.63	0.03 *	−0.17	0.22

**Legend:** An asterisk (*) represent a statistically significant value (*p* < 0.05).

**Table 2:** Effect of MAP 85 mmHg versus MAP 65 mmHg on the microcirculatory parameters and cardiac output in six sevoflurane anaesthetised Beagle dogs.

Microcirculatory Variables (Buccal) and Cardiac Output	Effect of MAP 85 mmHg Versus MAP 65 mmHg	
	Estimated Effect	*p*-Value
Proportion of perfused vessels (%)	−0.38	0.64
Proportion of perfused vessels < 20 µm (%)	−1.72	0.32
Perfused DeBacker density (mm/mm^2^)	−0.54	0.24
Perfused DeBacker density < 20 µm (mm/mm^2^)	0.02	0.97
Microvascular flow index (no unit)	0.01	0.79
Heterogeneity index (no unit)	−0.06	0.28
Cardiac output (L/minute)	−0.08	0.65

## Data Availability

Data on sublingual microcirculation is available in the Supplement Table (Supplementary Table S1).

## Supplementary Materials

The following supporting information can be downloaded at https://www.mdpi.com/article/10.3390/ani15121779/s1, Supplementary Table S1: Descriptive data of sublingual microvascular parameters in 6 sevoflurane anaesthetised Beagle dogs at the baseline and after the induction of endotoxemia with 1 mg/kg of *Escherichia coli* lipopolysaccharide intravenous.

## Abbreviations

The following abbreviations are used in this manuscript:

BL	baseline
CO	cardiac output
CO_2_	carbon dioxide
CRI	constant rate infusion
ECG	electrocardiogram
ET	endotoxin
EtCO_2_	end-tidal CO_2_
FB	fluid bolus
HI	heterogeneity index
HR	heart rate
LPS	lipopolysaccharide
MAP	mean arterial pressure
MFI	microvascular flow index
PaCO_2_	arterial partial pressure of CO_2_
pDBD	Perfused de Backer density
PmvCO_2_	mixed venous partial pressure of CO_2_
PPV	Proportion of perfused vessels
T	temperature
