# Supplementary material for: Effects of Endotoxemia and Blood Pressure on Microcirculation and Noradrenaline Needs With or Without Dexmedetomidine in Beagle Dogs—A Blinded Cross-Over Study
Source: Animals (Basel). 2025 Jun 17;15(12):1779. doi: 10.3390/ani15121779 (PMC12189173; doi:10.3390/ani15121779)
Supplement: Supplementary file 1 [file animals-15-01779-s001.zip › animals-3619302-supplementary.pdf]

Supp. Table S1: Descriptive data of sublingual microvascular parameters in 6 sevoflurane anaesthetised Beagle dogs at the baseline and after the induction of endotoxemia with 1 mg/kg of *Escherichia coli* lipopolysaccharide intravenous.

**Microvascular parameters**	**BL**	**ET**	**FB1**	**MAP65_1**	**MAP85_1**	**FB2**	**MAP65_2**	**MA85_2**
Proportion of perfused vessels (%)	92.7 (84-99.2)	91.3 (80.4-92.2)	92.1 (81.1- 94.1)					
Proportion of perfused vessels < 20 µm (%)	88.3 (73.9-98.5)	84.4 (76.8-93)	88.2 (84.2- 91.2)					
Perfused DeBacker density (mm/mm^2)	7.3 (7.0-11.6)	6.7 (5.8-8.9)	94.9514 92.94804 99.01961					
Perfused DeBacker density < 20 µm (mm/mm^2)	3.5 (2.7-4.3)	3.4 (1.7-5.8)	85.93935 76.83761 97.95263					
Microvascular flow index (no unit)	3 (0-3)	2.5 (0-3)	90.87303 58.80786 98.79636					
Heterogeneity index (no unit)	0 (0-0.1)	0.2 (0-1.9)	86.11559 81.13409 94.97395					

Legend: BL; baseline, ET; time after injection of 1 mg/kg of *Escherichia coli* lipopolysaccharide endotoxin intravenous. Values are presented as median (minimum-maximum).
